# The effect of antiseizure medication on mortality in spontaneous aneurysmal subarachnoid hemorrhage

**DOI:** 10.2478/jccm-2025-0014

**Published:** 2025-04-30

**Authors:** John Harold Kanter, Adam C. Glaser, Pablo Martinez-Camblor, Jakob V.E. Gerstl, Anna B. Lebouille-Veldman, Harshit Arora, Lauren Buhl, Myles D. Boone, Christopher S. Ogilvy

**Affiliations:** Department of Neurological Surgery, University of California, San Francisco, California; Brain and Spinal Injury Center, Zuckerberg San Francisco General Hospital, San Francisco, California, USA; Department of Neurological Surgery, Brigham and Women’s Hospital, Harvard Medical School Department of Neurological Surgery, Boston Children’s Hospital, USA; Department of Anesthesiology, Dartmouth Health Department of Biomedical Data Science, Geisel School of Medicine at Dartmouth, Dartmouth Hitchcock Medical Center, USA; Department of Neurosurgery, Computational Neuroscience Outcomes Center, Brigham and Women’s Hospital, Boston, USA; Department of Neurosurgery, Computational Neuroscience Outcomes Center, Brigham and Women’s Hospital, Boston, USA; Department of Neurosurgery, Leiden University Medical Center, Leiden, The Netherlands; Department of Neurosurgery, Computational Neuroscience Outcomes Center, Brigham and Women’s Hospital, Boston, USA. Department of Neurosurgery, University of Kentucky, Lexington, KY, USA; Department of Anesthesiology, Dartmouth Health, Dartmouth Hitchcock Medical Center, USA; Department of Anesthesiology, Dartmouth Health Department of Neurology, Dartmouth Health, Dartmouth Hitchcock Medical Center, USA; Neurosurgical Service, BIDMC Brain Aneurysm Institute, Harvard Medical School, Beth Israel Deaconess Medical Center, USA

**Keywords:** subarachnoid hemorrhage, seizure, mortality, antiseizure medication, seizure, death, MIMIC

## Abstract

**Background:**

Spontaneous aneurysmal subarachnoid hemorrhage (aSAH) is a major cause of morbidity and mortality in the United States. The efficacy of early antiseizure medication (ASM) is debated. Recent literature reports seizure rates ranging from 7.8% to 15.2% following spontaneous aSAH. Current guidelines recommend use of early ASM in patients with “high-risk features,” but whether early ASM use decreases the rate of death associated with aSAH remains unclear. This study assessed whether early administration of early ASM impacts mortality rates after spontaneous aSAH.

**Methods:**

We conducted a retrospective cohort study using a publicly available dataset from the Massachusetts Institute of Technology, Medical Information Mart for Intensive Care-III (MIMIC) database of all patients over the age of 18 with spontaneous aSAH resulting in an intensive care unit (ICU) admission to a major United States trauma center from 2001 to 2012. The primary exposure was receiving early ASM and primary outcome of death within 7 days. Different regression models were created to explore the association between early ASM administration within 24 hours of admission and a composite outcome of seizure and/or death within 7 days of admission. Secondary outcomes included 30-day and one-year mortality.

**Results:**

Of 253 patients with spontaneous aSAH, 148 received early ASM within 24 hours. Patients who did receive early ASM were less likely to die within 7 days of admission (adjusted odd ratio, [aOR]: 0.26 95% CI 0.10 to 0.68; P=0.006) but were more likely to have a seizure (aOR: 7.63 95% CI 2.07 to 28.17; P=0.002).

**Conclusion:**

Early ASM administration was associated with lower rates of death and composite death/seizure within 7 days of admission among patients who presented to an ICU with spontaneous aSAH. These findings suggest broader use of early ASM in patients who present with spontaneous aSAH may improve early mortality.

## Introduction

Spontaneous aneurysmal subarachnoid hemorrhage (aSAH) accounts for 5% of all strokes with an annual worldwide incidence of 6.1 per 100,000 person-years and is associated with significant morbidity and mortality [1,2]. The pathophysiology of neurologic injury after aSAH includes seizure activity and cortical spreading depression/depolarization (CSD), potentiating arteriolar vasoconstriction and delayed cerebral ischemia (DCI) [3]. The rates of seizure and CSD after aSAH are as high as 30% and 80%, respectively, and both are associated with unfavorable functional and cognitive outcomes morbidity and mortality [4,5].

Previous guidelines have recommended early antiseizure medication (ASM) (e.g., phenytoin) in the immediate post-hemorrhagic period but not beyond seven days post-hemorrhage [6]. The use of prophylactic phenytoin has been shown to be independently associated with worse cognitive outcomes after aSAH [7]. Early ASM has been shown to reduce seizure rates among a subset of patients with aSAH and high-risk features (i.e., ruptured middle cerebral artery (MCA) aneurysm, high-grade SAH, intracerebral hemorrhage (ICH), hydrocephalus, and cortical infarction; Class of Recommendation [COR] 2b, Level of Evidence [LOE] B-NR) [1].

Recent meta-analysis produced insufficient evidence to support the use of early ASM for primary or secondary prevention of clinical seizures following aSAH [8,9]. It remains unclear if the unfavorable cognitive outcomes with early ASM are related to DCI or phenytoin induced metabolic derangements [10,11].

More recent recommendations highlight technical advances in continuous video electroencephalographic (cvEEG) monitoring for patients with aSAH for the evaluation and management of sub-clinical epileptiform discharges and subsequent DCI (COR 2a, LOE B-NR) [1,12]. Additionally, newer generations of ASMs are associated with improvement in cognitive outcomes compared to prophylactic phenytoin—this effect may be due to the absence of poor outcomes seen with phenytoin, yet no difference in mortality. Thus, it remains unclear if expanded use of newer early ASMs might improve mortality outcomes [11]. To our knowledge, there is a paucity of literature evaluating the association of early ASM and mortality in patients with aSAH.

We report the results of a retrospective cohort study utilizing a well-established database of patients over the age of 18 who presented with spontaneous aSAH resulting in an intensive care unit (ICU) admission to a major US United States trauma center from 2001–2012 with exposure of early ASM administration and reporting a primary outcome of death within 7 days.

## Methods

This study utilized a publicly available dataset from the Massachusetts Institute of Technology, Medical Information Mart for Intensive Care-III (MIMIC) database [13]. The MIMIC database was approved by the Institutional Review Boards of the Massachusetts Institute of Technology and Beth Israel Deaconess Medical Center. Access to this database was granted to two of the authors (ACG and JHK). Further information about MIMIC is available at https://mimic.physionet.org/about/mimic/. The code to generate the raw dataset is available in the following GitHub repository https://github.com/adamcglaser/MIMIC_SAH_ASM_dataset. This study followed the Strengthening the Reporting of Observational Studies in Epidemiology (STROBE) reporting guidelines [14].

### Study Design

We assembled a retrospective cohort using the MIMIC (version 1.4) database, which consists of over 50,000 distinct hospital admissions for adult patients admitted to critical care units at Beth Israel Deaconess Medical Center between 2001 and 2012. The MIMIC database includes information such as patient demographics, International Classification of Diseases and Ninth Revision (ICD-9) codes, hourly vital signs, laboratory test results, procedures, medications, provider notes (admission notes, progress notes, transfer/discharge summaries), imaging reports, services involved, and mortality. Physiologic data was obtained from bedside monitors and validated by ICU nurses. Mortality data was combined with state records. Demographic data included age, gender, and ethnicity. Other admission data included hospital length of stay (LOS), intensive care unit length of stay (ICULOS), in-hospital mortality, and whether a patient was transitioned to comfort measures only (CMO). Baseline comorbidities were assessed using the Elixhauser comorbidity index [15]. ICU-based treatments such as initiation of mechanical ventilation (MV), vasopressor support, renal replacement therapy (RRT), external ventricular drain (EVD) placement, tracheostomy, and percutaneous endoscopic gastrostomy (PEG) tube placement were recorded. Subarachnoid hemorrhage severity was assessed by Hunt & Hess grade (HH) and modified Fisher grade (mFG) by manual chart review by three authors (J.V.E.G., A.B.L., and H.A.) [16,17]. Seizure events were manually assessed by chart review of provider notes and were coded in binary fashion as the “presence” or “absence” of seizure but were not otherwise defined as clinical or electrographic. Information regarding ASM administration was accessed via medication administration timing and provider notes. Administration of ASM was based on the discretion of the attending physician. Timing of ASM administration was precise to the day.

### Study Population

**Fig. 1.**
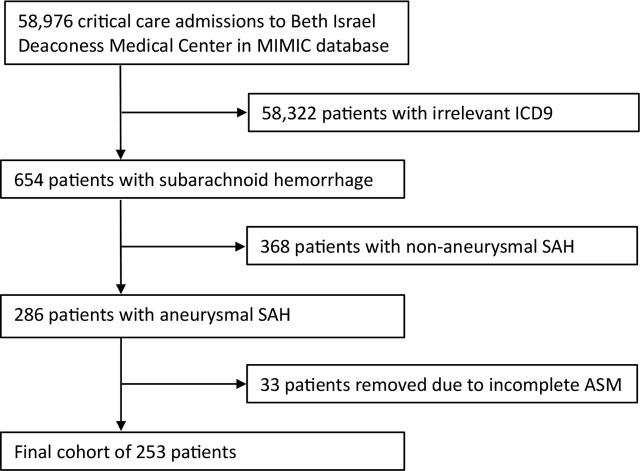
Flowchart demonstrating cohort criteria.

Our sample included all hospital admissions in the MIMIC database of patients over the age of 18 who were admitted to a medical ICU, critical care unit or surgical ICU with a diagnosis of ruptured aSAH. Diagnoses were determined by ICD9 codes associated with individual hospital admissions (see supplementary file for ICD9 codes). ASMs included were carbamazepine (Tegretol), fosphenytoin, lacosamide (Vimpat), lamotrigine (Lamictal), levetiracetam (Keppra), oxcarbazepine (Vimpat), phenobarbital, phenytoin, pregabalin (Lyrica), divalproex sodium (Depakote), and valproic acid (Depakene). Exclusion criteria included: admissions with incomplete drug information, re-admissions with a primary ICD9 code of aSAH, patients who received ASMs after 24 hours of hospital admission. Figure 1 depicts the details of the final study population.

### Primary Exposure

The primary exposure early ASM administration (within 24 hours of admission) in patients presenting with spontaneous ruptured aSAH.

### Primary Outcome

The primary outcome was death within the first seven days of admission to the ICU.

### Secondary Outcome

Secondary outcomes include seizure within the first seven days, composite seizure or death within first seven days, 30-day and one-year mortality.

### Statistical Analysis

Continuous variables were reported as median, first and third quartiles. Wilcoxon test was used for comparing the location parameters between groups. Absolute and relative frequencies summarized categorical variables, and Chi-squared test was used for comparisons. Resultant odds ratios (ORs) and 95% confidence intervals for four different logistic regression models were reported as association measures between the binary outcomes and the exposure. The initial unadjusted model reports the OR for death, seizure, and composite outcome (death or seizure) associated with early ASM administration. Model 1 adjusts for demographic variables (age, gender, and ethnicity). Model 2 accounts for a priori prespecified covariates based on biological plausibility (Elixhauser [van Walraven], GCS, mechanical ventilation, HH, and mFG). In addition, results from doubly robust inverse propensity weighing (DR-IPW) models are also provided. DR-IPW combines both the inverse propensity score weighting (IPSW) and adjusts by the propensity score variables. This procedure allows for the comparison of similar patients from similar populations. Variables included in the risk-profile model (Model 2) are also included in the propensity score model. With a similar approach, we provided hazard ratios and 95% confidence intervals derived from proportional hazards Cox regression models for modeling the time to death. We considered a complete case analysis and patients with all required variables were included. Subsequently, in the multivariate models, missing values in covariates with less than 5 missing cases were replaced by the overall median. Two-sided P-values are provided. All statistical analyses were conducted in R version 3.3.1 (Vienna, Austria) [18].

## Results

A total of 253 patients admitted to an ICU with aSAH were included in the analysis (Figure 1). Baseline characteristics of this cohort are shown in Table 1. Among all patients, the median age [1^st^ and 3^rd^ quartiles] was 56 [47–69] years. Patients were predominantly white (165 patients, 65.2%), and female (165, 65.2%). There were no differences in baseline demographic or comorbidity measures between the prophylaxis and no prophylaxis groups. Admission GCS was categorized according to the World Federation of Neurological Societies (WFNS). Patients receiving early ASM presented with lower GCS [quartiles] (11 [14–15] vs. 15 [13–15], P = 0.052). There was no difference in admission HH score between the two groups, suggesting equal clinical SAH severity (P=0.64). The majority of patients (186, 73.5%) were admitted with anterior circulation aneurysms and predominantly treated by endovascular coiling (176, 69.6%) or open surgical clipping (51, 20.2%). There were 26 patients with missing data regarding treatment modality. Of this cohort, there were 148 (58.5%) patients who received early ASM within 24 hours of admission. Patients who did receive ASM were less likely to experience the composite outcome than those who did not (32 (21.8%) vs 28 (26.7%); P = 0.45) (Table 1).

**Table 1.** Demographics of the cohort; early (pASM) and no drug administration (No ASM) groups, including age, sex, race, GCS on admission, ICD9 diagnosis, and Elixhauser van Walraven comorbidity score.

	**No ASM (N=105)**	**Early ASM (N=148)**	**SMD**	**p-Value**
Age, median [Quartiles]	56 [47–73]	56 [47–67]	0.08	0.657
Gender, Female, N (%)	68 (64.8)	97 (65.5)	−0.02	1

Race, N (%)				
White	65 (61.9)	100 (67.6)	−0.12	0.425
Black	7 (6.7)	16 (10.8)	−0.15	0.364
Latino	8 (7.6)	7 (4.7)	0.12	0.491

GCS at admission, median [Quartiles]	15 [13–15]	14 [11–15]	0.16	0.052
GCS 15, N (%)	54 (51.9)	58 (39.2)	0.26	0.061
GCS 14, N (%)	20 (19.2)	33 (22.3)	−0.07	0.666
GCS 13, N (%)	10 (9.6)	14 (9.5)	0.01	1
GCS (7–12), N (%)	10 (9.6)	28 (18.9)	−0.27	0.064
GCS (3–6), N (%)	10 (9.6)	15 (10.1)	−0.02	1

HH, N (%)				
I	21 (20.0)	25 (16.9)	0.08	0.641
II	36 (34.3)	57 (38.5)	−0.09	0.579
III	19 (18.1)	32 (21.6)	−0.09	0.596
IV	7 (6.7)	11 (7.4)	−0.03	1
V	21 (20.0)	23 (15.5)	0.12	0.451

mFG, N (%)				
I	33 (31.4)	28 (18.9)	0.29	0.032
II	7 (6.7)	29 (19.6)	−0.39	0.007
III	14 (13.3)	23 (15.5)	−0.06	0.757
IV	49 (46.7)	68 (45.9)	0.01	1
Elixhauser, median [Quartiles]	2 [1–3]	2 [1–3]	0.04	0.702
Anterior Circulation	74 (70.5)	112 (75.7)	−0.13	0.436
Posterior Circulation	24 (22.9)	31 (20.9)	0.06	0.835

Aneurysm Treatment Modality				
Clip	17 (17.4)	34 (23.9)	−0.16	0.286
Coil	77 (78.6)	99 (69.7)	0.20	0.169
Aneurysmal Re-bleeding	3 (2.9)	7 (4.7)	−0.10	0.67
Vasospasm	26 (24.8)	48 (32.4)	−0.17	0.237

Other Interventions, N (%)				
Tracheostomy	2 (1.9)	8 (5.4)	−0.19	0.28
Percutaneous endoscopic gastrostomy tube	4 (3.8)	13 (8.8)	−0.20	0.193
Ventriculostomy	9 (8.6)	18 (12.2)	−0.12	0.481
CSF Shunt	3 (2.9)	11 (7.43)	−0.21	0.197
RRT, N (%)	1 (0.95)	1 (0.7)	0.03	1
Mechanical Ventilation, N (%)	74 (70.5)	101 (69.2)	0.03	0.935

Discharge at, N (%)				
Home	34 (32.4)	44 (29.7)	0.06	0.755
Rehabilitation	21 (20.0)	40 (27.0)	−0.17	0.255
Dead (in Hospital)	32 (30.5)	23 (15.5)	0.36	0.007
Other	18 (17.1)	41 (27.7)	−0.25	0.071

ASM, antiseizure medication, CSF, cerebrospinal fluid, GCS, Glasgow Coma Scale, HH, Hunt & Hess grade, ICD9, International Classification of Diseases Ninth Revision, mFG, modified Fisher Grade, RRT, Renal replacement therapy, SAH, subarachnoid hemorrhage, SMD, standardized mean difference

Table 2 shows the observed number and percentage of outcomes including unadjusted ORs and 95% confidence intervals by group (No ASM/Early ASM) overall and for different subgroups, based on presentation severity stratified by GCS, HH, or mFG to examen if these presenting factors had an association with the observed outcome. Overall, 25 (9.9%) patients had a seizure, 3 (2.9%) in the non-prophylaxis group, and 22 (15.0%) in the early ASM group (P = 0.003). There were 39 deaths within 7 days from admission. Of the patients that died, 25 (23.8%) were in the non-prophylaxis group, and 14 (9.5%) were in the early ASM group (P = 0.003). Notably, more patients not receiving ASM died in the hospital (32, 30.5% vs 23, 15.5%; P = 0.007) (Table 1).

**Table 2.** Observed number of outcomes by (No ASM/pASM) groups and by different sensitivity groups at 7 days. OR [95% CI]: Unadjusted odd ratio and 95% confidence intervals.

	**No ASM**	**Early ASM**	**OR [95% CI]**	**p-Value**
Total	105	148		
Seizure	3 (2.9)	22 (15.0)	5.98 (1.7 to 20.6)	0.003
Death	25 (23.8)	14 (9.5)	0.33 (0.16 to 0.68)	0.003
Composite	28 (26.7)	32 (21.8)	0.77 (0.43 to 1.4)	0.453

GCS 15-14	74	91		
Seizure	1 (1.4)	15 (16.5)	14.6 (1.88 to 113.4)	0.003
Death	12 (16.2)	7 (7.7)	0.44 (0.16 to 1.17)	0.144
Composite	13 (17.6)	18 (19.8)	1.17 (0.53 to 2.59)	0.872

GCS 3–13	30	57		
Seizure	2 (6.7)	7 (12.5)	2.04 (0.40 to 10.5)	0.636
Death	12 (40)	7 (12.3)	0.18 (0.06 to 0.55)	0.007
Composite	14 (46.7)	14 (15.1)	0.35 (0.14 to 0.91)	0.032

HH: I, II	57	82		
Seizure	2 (3.5)	11 (13.4)	4.32 (0.92 to 20.3)	0.094
Death	1 (1.7)	1 (1.2)	0.70 (0.04 to 11.4)	1.00
Composite	3 (5.3)	11 (13.4)	2.83 (0.75 to 10.6)	0.199

HH: III, IV, V	48	66		
Seizure	1 (2.1)	11 (16.7)	9.55 (1.19 to 76.8)	0.026
Death	24 (50.0)	13 (19.7)	0.24 (0.10 to 0.55)	0.001
Composite	25 (52.1)	21 (31.8)	0.44 (0.20 to 0.94)	0.055

mF: I, II, III	56	80		
Seizure	1 (1.8)	11 (13.8)	8.90 (1.11 to 71.1)	0.035
Death	4 (7.1)	4 (5.0)	0.51 (0.11 to 2.4)	0.879
Composite	5 (8.9)	14 (17.5)	2.01 (0.67 to 6.0)	0.243

mF: IV	49	68		
Seizure	2 (4.1)	11 (16.2)	4.6 (0.97 to 21.8)	0.075
Death	21 (42.9)	10 (14.7)	0.25 (0.10 to 0.59)	0.001
Composite	23 (46.9)	18 (26.5)	0.44 (0.14 to 0.97)	0.042

ASM, antiseizure medication, GCS, Glasgow Coma Scale, HH, Hunt & Hess grade, mFG, modified Fisher Grade, OR, odds ratio

Table 3 displays ORs and 95% confidence intervals at 7 days for seizure, death, and the composite outcome of seizure and/or death for the non-prophylaxis group vs. the early ASM group. An unadjusted model found a higher OR for seizure (5.98 [1.74 to 20.56]; P = 0.004), lower OR of death (0.33 [0.16 to 0.68]; P = 0.002), and similar OR for the composite outcome of death and/or seizure (0.77 [0.43 to 1.37]; P = 0.37). Model 1 was adjusted for demographic factors: gender, age, and ethnicity and showed a higher OR for seizure (6.64 [1.86 to 23.66]; P = 0.003), a lower OR for death (0.35 [0.17 to 0.72]; P = 0.005), and similar OR for the composite outcome (0.79 [0.44 to 1.41]; P = 0.42). Model 2 additionally adjusts for critical care and aSAH factors: Elixhauser comorbidity index, GCS, mechanical ventilation, HH grade, and mFG and found a higher OR for seizure (7.63 [2.07 to 28.17]; P = 0.003), a lower OR for death (0.26 [0.10 to 0.68]; P = 0.006), and similar OR for the composite outcome (0.79 [0.40 to 1.55]; P = 0.498). Lastly, a DR-IPSW model found a higher OR for seizure (7.65 [2.03 to 28.89]; P = 0.003), a lower OR for death (0.25 [0.09 to 0.67]; P = 0.006), and similar OR for the composite (0.79 [0.40 to 1.57]; P = 0.51) for the early ASM group vs the non-prophylaxis group.

**Table 3.** Regression models and adjusted odds ratios at 7-days for outcomes: seizure, death, and composite if foregoing ASM administration. Results are based on unadjusted data, and adjusted models

	**Seizure**				**Death**				**Composite**			

	**OR**	**[95% CI]**		**p-Value**	**OR**	**[95% CI]**		**p-Value**	**OR**	**[95% CI]**		**p-Value**
Unadjusted	5.98	1.74	20.56	0.0045	0.33	0.16	0.68	0.0025	0.77	0.43	1.37	0.3688
Model 1	6.64	1.86	23.66	0.0035	0.35	0.17	0.72	0.0048	0.79	0.44	1.41	0.4207
Model 2	7.63	2.07	28.17	0.0023	0.26	0.10	0.68	0.0063	0.79	0.40	1.55	0.4880
DR-IPSW	7.65	2.03	28.89	0.0027	0.25	0.09	0.67	0.0062	0.79	0.40	1.57	0.5062

**Model 1:** Unadjusted + Gender + Age + Race (White); **Model 2:** Model 1 + Elixhauser + GCS + Mechanical ventilation + HH + mFG.

**DR-IPSW:** PS based on Model 2 variables + same adjustment; ASM, antiseizure medication, GCS, Glasgow Coma Scale, HH, Hunt & Hess grade, mFG, modified Fisher Grade, OR, odds ratio

Table 4 displays hazard ratios (HR) derived from proportional hazard Cox regression models based on similar unadjusted, adjusted, and DR-IPW approaches.

**Table 4.** Survival Models – Hazard ratios at 7-days, 30-days, and 1-year

	**7 Days**				**30 Days**				**1 Year**			

	**HR**	**[95% CI]**		**p-Value**	**HR**	**[95% CI]**		**p-Value**	**HR**	**[95% CI]**		**p-Value**
Unadjusted	0.38	0.20	0.72	0.0034	0.78	0.59	1.03	0.0791	0.84	0.65	1.10	0.2040
Model 1	0.42	0.21	0.80	0.0092	0.78	0.59	1.03	0.0847	0.84	0.65	1.09	0.1990
Model 2	0.33	0.16	0.66	0.0021	0.73	0.55	0.98	0.0332	0.79	0.60	1.03	0.0831
DR-IPSW	0.32	0.15	0.69	0.0036	0.73	0.55	0.97	0.0285	0.79	0.60	1.03	0.0823

**Model 1:** Unadjusted + Gender + Age + Race (White); **Model 2:** Model 1 + Elixhauser + GCS + Mechanical ventilation + HH + mFG. **DR-IPSW:** PS based on Model 2 variables + same adjustment.

ASM, antiseizure medication, GCS, Glasgow Coma Scale, HH, Hunt & Hess grade, HR, hazard ratio, mFG, modified Fisher Grade.

**Fig. 2.**
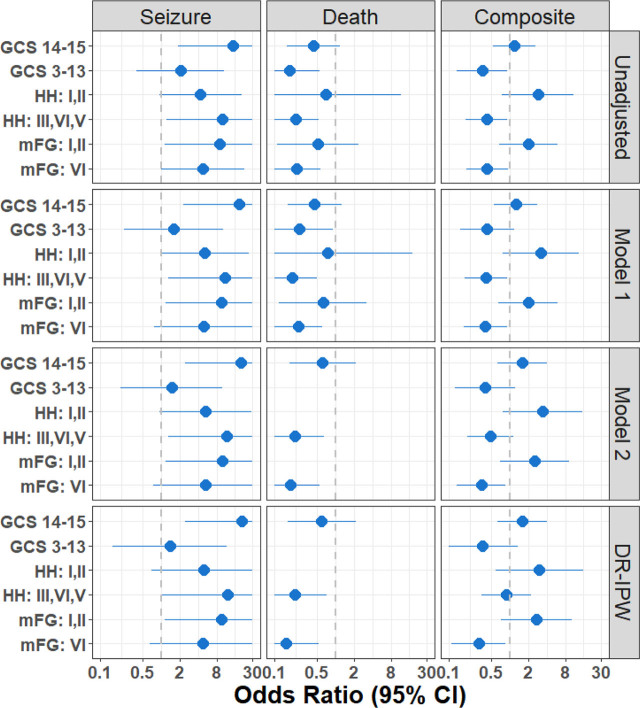
**Forest plot of sensitivity analysis. Data associated with this figure is provided in Table 2 (unadjusted OR) as supplementary material (Table S3).** ASM, antiseizure medication, GCS, Glasgow Coma Scale, HH, Hunt & Hess grade, mFG, modified Fisher Grade, OR, odds ratio.

Forest plot in Figure 2 shows a sensitivity analysis including ORs for seizure, death, and the composite outcome of seizure or death based on the unadjusted, adjusted, and DR-IPW models stratified by GCS, HH, and mFG again to examine if these presenting factors had an association with the observed outcome. The ORs for excess death or seizure for patients who did not receive early ASM are consistent across all models.

**Fig. 3.**
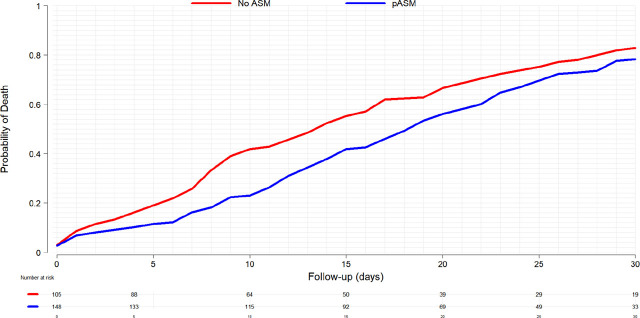
Kaplan-Meier curve for the probability of death within the first 30-days

Figure 3 shows the Kaplan-Meier estimation for the probability of death within the first 30-days after admission demonstrating the reduction in mortality at 7 days. This effect appears to decrease at 30-days.

Supplemental Table S1 shows additional descriptive variables. Patients who received early ASM were more likely to have mFG II aSAH (P = 0.007), while patient not receiving early ASM were more likely to present with mFG I aSAH (P = 0.032).

Supplementary Table S2 shows medical comorbidities stratified by exposures to early ASM and shows no difference between groups. Additionally, Table S4 shows OR by location of aneurysm (anterior versus posterior circulation) and demonstrates no difference in outcomes.

## Discussion

This large, retrospective cohort study demonstrates that early ASMs are associated with reduced mortality in the first 7 days following ICU admission in patients presenting with spontaneous aSAH, yet this effect is diluted at 30 days. The significant effect of early ASM administration on mortality is consistent regardless of model adjustments for demographics, Elixhauser comorbidity scores, presenting GCS, mechanical ventilation, and SAH severity at presentation. This finding suggests that broader administration of early ASM, regardless of presenting patient characteristics, may be beneficial. This study adds to our existing understanding by demonstrating a significant association between early ASM administration and reduced mortality in the first 7 day following ICU admission among spontaneous aSAH patients at all levels of severity.

The 2023 Guidelines for the Management of Patients with Aneurysmal Subarachnoid Hemorrhage from the American Heart Association and American Stroke Association noted that the impact of ASM administration in a “targeted and time-limited manner” is not well defined, and there are currently no blinded, randomized controlled trials to support recommendations [1].

A 2013 Cochrane review found no relevant randomized or quasi-randomized controlled trials evaluating early ASM versus placebo in aSAH [19]. Of the eight studies that were screened and excluded, five were retrospective evaluations of seizure rates with early ASMs, one was a prospective study allocated to short-term (three days) or long-term (until discharge) phenytoin treatment, one evaluated cognitive disability associated with phenytoin exposure in aSAH, and one was a comparison between levetiracetam and phenytoin in patients with traumatic brain injury (TBI) [20,21,22].

There is evidence demonstrating a mortality benefit of early ASM administration in older adults (age 65 and older) with TBI, suggesting that early ASM may provide a mortality benefit in other conditions where patients are predisposed to seizures, non-convulsive status epilepticus (NCSE), and DCI [23].

The finding that the early ASM group had a higher OR for seizure when compared to the group not receiving early ASM may indicated that ASM administration was intended for treatment of seizure or seizure-like activity seen at presentation rather than true prophylaxis. Unfortunately, this dataset does not have the granularity to allow for establishing a clear timeline between ASM administration and seizure, therefore due to this limitation we chose to select mortality as primary outcome.

The loss of mortality benefit in this cohort may be multifactorial. These data are older and may reflect practices from a previous generation of SAH guidelines [6]. The use of cvEEG following aSAH has been shown to detect subclinical seizures and NCSE and be predictive predict DCI before it may be clinically evident. NCSE is associated with high mortality and morbidity, and current guidelines recommend the use of cvEEG for detection (COR 2a; LOE B-NR) [1,24]. We are also unable to infer the clinical decision making of individual practitioners and their use of early ASM.

This retrospective cohort study followed the STROBE criteria; however, it has several limitations [14]. It is a single-center study at a large academic hospital, which limits generalizability. Models adjusting for demographic, comorbidity, and critical care differences, however, found the same significant benefit of early ASM. As mentioned previously, a major limitation of this dataset is the inability to establish timing of early ASM administration in relation to seizure activity hindering the biological interpretation of these events, therefore we selected mortality as the primary outcome. The trade-off is that direct comparison to other studies is more challenging. This study also did not account for the use of benzodiazepines or sedating medications like propofol that may alter seizure thresholds. As this study only includes aSAH patients admitted to the ICU, we cannot comment on patients that were not admitted to an ICU (i.e., patients that died in the emergency department); although, standard practice, even in cases with a seemingly poor prognosis, are typically admitted to an ICU. The MIMIC database did not include data beyond the ICU stay so we have no data on the effects of early administration of early ASMs on late seizures. The MIMIC database itself, nor do we have long term follow-up to assess neurologic outcomes (e.g., modified Rankin scale, Glasgow Outcomes Scale Extended, or other cognitive function measures). The MIMIC database has certain limitations as well, including the fact that drugs administered in the ED are not recorded and that oral drug administration time is precise only to the date of administration, requiring patients in whom the chronological order of oral ASM administration and seizure occurrence could not be determined to be excluded. While these are important limitations, the consistency of the effect of early ASM on the primary outcome across multiple models that include age, demographics, and comorbidity scores suggests that their impact was limited and increases the generalizability of our findings.

## Conclusion

Our findings support the use of early ASM in patients presenting with spontaneous aSAH and highlight the need for prospective, randomized controlled trials evaluating the use of early ASM in this patient population, including the use of cvEEG guiding management.
